# Rapid isolation of pan-neutralizing antibodies against Omicron variants from convalescent individuals infected with SARS-CoV-2

**DOI:** 10.3389/fimmu.2024.1374913

**Published:** 2024-03-06

**Authors:** Peng Yu, Jingping Ran, Ruiqi Yang, Hang Zhu, Song Lu, Yuzhang Wu, Tingting Zhao, Tianchen Xiong

**Affiliations:** ^1^ Antibody Research Platform, Chongqing International Institute for Immunology, Chongqing, China; ^2^ School of Pharmacy and Bioengineering, Chongqing University of Technology, Chongqing, China

**Keywords:** SARS-CoV-2, COVID-19, Omicron, neutralizing antibody, beacon

## Abstract

**Introduction:**

The emergence of SARS-CoV-2 Omicron subvariants has presented a significant challenge to global health, as these variants show resistance to most antibodies developed early in the pandemic. Therapeutic antibodies with potent efficacy to the Omicron variants are urgently demanded.

**Methods:**

Utilizing the rapid antibody discovery platform, Berkeley Lights Beacon, we isolated two monoclonal neutralizing antibodies, 2173-A6 and 3462-A4. These antibodies were isolated from individuals who recently recovered from Omicron infections.

**Results:**

Both antibodies, 2173-A6 and 3462-A4, demonstrated high affinity for the RBD and effectively neutralized pseudoviruses from various Omicron lineages, including BA.4/5, XBB.1.16, XBB.1.5, and EG.5.1. This neutralization was achieved through binding to identical or overlapping epitopes.

**Discussion:**

The use of the Beacon platform enabled the rapid isolation and identification of effective neutralizing antibodies within less than 10 days. This process significantly accelerates the development of novel therapeutic antibodies, potentially reducing the time required to respond to unknown infectious diseases in the future.

## Introduction

The coronavirus disease 2019 (COVID-19) pandemic caused by severe acute respiratory syndrome coronavirus 2 (SARS-CoV-2) has resulted in great damage to public health (1). The surface spike glycoprotein (S) of SARS-CoV-2 plays a crucial role in cell entry by interacting with the angiotensin-converting enzyme 2 (ACE2) receptor on the cell surface through its receptor-binding domain (RBD) (2). Since the identification of SARS-CoV-2, a series of variants of concern (VOCs) such as Alpha (B.1.1.7) (3, 4), Beta (B.1.351) (5, 6), Gamma (P.1) (7, 8) and Delta (B.1.617.2) (9, 10) have emerged, exhibiting increased virus infectivity and immune evasion. Notably, in November 2021, a novel VOC named Omicron (B.1.1.529) was first identified in Botswana and South Africa. It quickly replaced Delta within weeks, becoming the dominant variant in several countries owing to its enhanced transmissibility (11–15).

Each VOC carries mutations across the SARS-CoV-2 genome, with particular concern for those within the S protein. Previous studies on SARS-CoV-2 variants have revealed that the S protein serves not only as the site binding to the host receptor ACE2 but also as the essential target for therapeutic monoclonal antibodies (mAbs) and neutralizing antibodies produced by the natural and vaccine-induced immune response (16–18). While VOCs like Alpha, Beta, Gamma and Delta exhibit 9 to 12 mutations within the S protein, impacting the effectiveness of therapeutic mAbs to a certain extent (19–23). Omicron variants are characterized by more than 35 substitutions in the S protein, with 15 occurring in the RBD. This raises concerns about the effectiveness of currently approved antibodies for clinical use (14, 24, 25).

Monoclonal antibodies have gained significant interest as potential prophylactic and therapeutic measures against viral infections due to their desirable qualities, including high specificity and their capacity to boost immune responses (26–28). The widespread transmission of SARS-CoV-2 has led to extensive efforts in developing mAbs targeting the RBD of the S protein. As a result, several neutralizing mAbs have been approved under an Emergency Use Authorization (EUA) for the early treatment of COVID-19 (29–33). Despite these advancements, the Omicron variants, characterized by intense mutations in the RBD of the S protein, have shown significant resistance to the majority of approved neutralizing mAbs for clinical use (34–38). This poses a pressing need for the identification of neutralizing mAbs that can effectively combat the currently prevalent Omicron variants and potential newly emergent VOCs. Such discoveries are essential for the continuous development of robust and durable therapeutics for COVID-19.

Monoclonal antibodies employed in therapeutics are primarily derived from *in vivo* immunization, a process often succeeded by either hybridoma immortalization or the application of immune cell libraries for display technologies like phage or yeast (39–41). While these methods are significant and reliable, they come with notable limitations. Hybridoma approaches, requiring the immortalization of antibody-secreting cells (ASCs) through somatic fusion with a myeloma cell line, commonly face challenges, such as a reduced survival rate leading to a loss of target cell clones. Furthermore, mAbs obtained through display technologies exhibit lower affinity due to their origin from random pairings of immunoglobulin variable heavy (VH) and variable light (VL) regions. In addition, both hybridoma and display technologies require substantial labor costs, thus extending the timeline for novel therapeutic antibody development (39–41).

Neutralizing monoclonal antibodies (mAbs) sourced from humans hold great promise as therapeutic agents against emerging viruses, primarily due to their safety profile when contrasted with antibodies obtained from immunized animals, whose immunoglobulins may pose antigenic risks to humans (26, 42). Additionally, samples collected from convalescent individuals with prior viral infections present a rich and accessible reservoir of mAbs for therapeutic development (43). Since the emergence of SARS-CoV-2, numerous research groups have reported the direct isolation of neutralizing antibodies from survivors. This process involves Fluorescence-Activated Cell Sorting (FACS)‐based enrichment of antigen-specific memory B cells, followed by the identification of the mAbs they generate (44–55). However, an immediate distinction among these cells to identify those with the potential to secrete neutralizing antibodies remains challenging, and this determination requires further, labor-intensive assays.

Recently, innovative approaches utilizing microfluidic technologies for direct B cell antibody discovery have been described (42, 56–58). One noteworthy technology platform, developed by Berkeley Lights, is the Beacon platform. This platform integrates opto-electropositioning (OEP), microfluidics, and microscopy to enable single-cell manipulation, culturing, and phenotypic analysis on nanofluidic chips (59–62). A significant breakthrough lies in the platform’s capability to directly identify single antibody-secreting cells (ASCs) on chips, highlighting not only antigen specificity but also additional attributes like the ability to neutralize the Receptor Binding Domain (RBD) of the SARS-CoV-2 Spike (S) protein. After the high-throughput screening, ASCs of interest could be exported individually off the microfluidic chip for retrieving sequences encoding native VH and VL pairings of the original antibodies (59–62).

In this study, we utilized the Beacon platform to isolate mAbs recognizing the RBD of S protein from individuals with a history of Omicron infection, and we identified two promising candidates, namely 2173-A6 and 3462-A4. Both 2173-A6 and 3462-A4 exhibited robust RBD binding and demonstrated potent neutralizing capacities against pseudoviruses representing various Omicron lineages, including BA.4/5, XBB.1.16, XBB.1.5 and EG.5.1. Overall, the Beacon platform facilitated the rapid isolation and identification of potential neutralizing antibodies from human sources within a remarkable 10-day timeframe. This efficiency will substantially streamline downstream workflows and expedite the development of innovative therapeutic antibodies, not only for addressing newly emerging SARS-CoV-2 variants but also for combating other unknow infectious diseases in the future.

## Materials and methods

### Human sample collection

Blood samples were collected from COVID-19 patients during their convalescence, with the time between symptom onset and sample collection averaging around 50 days. Two adult participants served as healthy donors for this study. The samples were collected in cell preparation tubes containing sodium citrate (BD Bioscience). Subsequently, peripheral blood mononuclear cells (PBMCs) were isolated from the blood samples using Ficoll (Cytiva, 10308255). After washing with PBS, the PBMCs were suspended in a serum-free cryopreserved solution (Pricella, PB180438), frozen in a freezing chamber at −80°C, and then transferred to liquid nitrogen for long-term preservation. The study received approval from the Institutional Review Board (IRB) of Chongqing International Institute for Immunology, and all participants provided written informed consent.

### Cell lines, recombinant proteins and viruses

HEK293-ACE2 cells (Vazyme, DD1401) were cultured in OPM-293 CD05 Medium (Opmbiosciences, 81075-001) and maintained at 37°C in a humidified atmosphere with 5% CO2. Additionally, HEK293F cells (obtained from Kairuibiotech) were cultured in KOP293 expression medium (Kairuibiotech, K03252) under the same conditions. Recombinant proteins, including ACE2 (10108-H08H), Spike (BA.4/5) S1+S2 trimer (40589-V08H32), and Spike RBDs of BA.4/5 (40592-V08H130), XBB.1.5 (40592-V08H146), XBB.1.16 (40592-V08H136), and EG.5.1 (40592-V08H151), were purchased from Sino Biological. RBD recombinant proteins were biotinylated following the protocol of the EZ-Link™ NHS-PEG4-Biotin Kit (Thermo Fisher Scientific, A39259). Pseudoviruses, representative of wild-type SARS-CoV-2 and its variants (B.1.1.529, BA.4/5, XBB.1.5, XBB.1.16, EG.5.1), were acquired from Vazyme company.

### RBD-specific memory B cells enrichment and plasmablast activation

B cells were isolated from PBMCs of convalescent individuals with COVID-19 using immunomagnetic negative selection (EasySep Human B Cell Enrichment Kit, STEMCELL, 17954). In this process, Tetrameric Antibody Complexes and dextran-coated magnetic particles targeted and removed non-B cells. Following isolation, B cells underwent staining with anti-CD19-APC (BioLegend, 302212), anti-IgD-FITC (BioLegend, 348206), anti-IgM–FITC (BioLegend, 314506) phenotyping antibodies, and biotinylated SARS-CoV-2 (BA.4/5) Spike RBD antigen. Viability was assessed using 7-AAD Stain (Invitrogen, 00699342). Class-switched memory B cell-antigen complexes (CD19^+^Ag^+^IgM^-^IgD^-^7-AAD^-^) were then detected with a PE-labeled streptavidin conjugate (BioLegend,127807), and target memory B cells were isolated via flow-cytometric sorting using a BD FACSAira Fusion (BD Biosciences). Subsequently, cells were cultured and activated in human B cell expansion medium for 8 days (ImmunoCultTM Human B Cell Expansion Kit, STEMCELL, 1000645). Flow-cytometric data analysis was conducted using FlowJo version 10.8.1 (Tree Star).

### Single RBD-specific ASCs selection by Berkeley lights beacon system

Activated ASCs samples were imported automatically onto OptoSelect^TM^ 11k chips in a novel plasmablast survival medium that promotes antibody secretion and preserves cell viability (63, 65). (BerkeleyLights, 75002051). Subsequently, single-cell penning was executed through OEP technology, employing light for the precise transfer of B cells into individual nanoliter-volume chambers known as NanoPens. This light-based manipulation enabled the transfer of thousands of ASCs into pens across multiple chips in each workflow. To identify antibodies binding to the SARS-CoV-2 (BA.4/5) Spike RBD protein, we conducted an on-chip, fluorescence-based assay. Conjugated beads were prepared by coupling biotinylated RBD (BA.4/5) protein to streptavidin-coated assay beads (Berkeley Lights, 520-00053). The assay involved mixing these beads with a fluorescently labeled anti-human secondary antibody (AF568, Thermo Fisher, A-21090) at a 1:100 dilution. This mixture was then imported into OptoSelect 11k chips. Antigen-specific antibodies bound to the RBD (BA.4/5)-conjugated beads, sequestering the fluorescent secondary antibody. NanoPens adjacent to the fluorescent beads were identified as containing cells secreting antigen-specific antibodies. Another on-chip assay was conducted to select antibodies that could block the interaction between hACE2 and the RBD of SARS-CoV-2 (BA.4/5). This blocking assay involved co-incubating ASCs and RBD-conjugated streptavidin-coated assay beads (Berkeley Lights, 520-00053) in NanoPen chambers. Subsequently, a mixture of fluorescently labeled hACE2 (AF647, Thermo Fisher, A330009) and an anti-Human IgG (H+L) antibody (AF568, Thermo Fisher, A-21090) at a 1:1000 dilution was perfused through the OptoSelectTM 11k chip. Antibodies with RBD binding and RBD-hACE2 blocking activities were identified by locating NanoPen chambers with fluorescence in both the Beacon TRED filter cube and Cy5 filter cube. Antigen-specific cells of interest were exported from specific NanoPen chambers to individual wells of 96-well reverse transcription-PCR plates containing lysis buffer (Qiagen, 1070498).

### Single B cell sequence and plasmids construction

Following exportation from the Beacon system, heavy- and light-chain sequences of antibodies secreted by B cells binding to RBD (BA.4/5) were amplified and recovered using components of the Opto Plasma B Discovery cDNA Synthesis Kit (Berkeley Lights, 750-02030). In a concise procedure, RNA from a single B cell underwent purification and isolation using Agencourt RNAClean XP Beads (Beckman Coulter, A63987). The eluted RNA was then subjected to a 9 uL RT (reverse transcription) reaction. Subsequently, the first-strand cDNA synthesis and total cDNA amplification were carried out according to the manufacturer’s protocol (Berkeley Lights, 750-02030). The resulting total cDNA was purified using Agencourt AMPure XP Beads (Beckman Coulter, A63881) and made ready for sequencing through the Opto Plasma B Discovery Sanger Prep Kit (Berkeley Lights, 750-02041). The nucleotide sequence from amplicons was determined via Sanger sequencing in reverse orientation. The obtained data were subjected to analysis in Geneious Biologics, utilizing the single clone antibody analysis pipeline for antibody annotation.

### MAbs expression and purification

The paired sequences of heavy and light chains for the selected monoclonal antibodies (mAbs) underwent codon optimization, synthesis (Tsingke Biotech), and cloning into separate mammalian expression vectors (pcDNA™ 3.4 TOPO™, A14697) containing the IgG1 constant regions of humans. Subsequently, these vectors were co-transfected into HEK293F cells using the TA293 transfection reagent (kairuibiotech, K20001) in KPM serum-free medium (kairuibiotech, K03125). After a 5-day incubation period, cell culture supernatants were collected and subjected to purification using a Protein A column (smart-lifesciences, SA012100). The bound mAbs were washed with a buffer containing 20 mM Na_2_HPO_4_, 150 mM NaCl (pH 7.0), eluted with 0.1 M acetic acid (pH 3.0-4.5) on an ÄKTA pure 150 (Cytiva) system, and resuspended into PBS through centrifugation using 30 kDa MWCO membrane centrifugal filter units (Millipore, UFC903024). The purified antibodies were validated through SDS-PAGE and stored at -80°C.

### ELISA

ELISA plates (LABSELECT, 31111) were coated with SARS-CoV-2 RBD proteins (BA.4/5, XBB.1.5, XBB.1.16, and EG.5.1) at a concentration of 1 ug/mL at 4°C overnight. Subsequently, the plates underwent blocking with 2% defatted milk in DPBS containing 0.05% Tween-20 (DPBS-T) for 1 hour. Following the blocking step, recombinant monoclonal antibodies (mAbs) or human serum samples were added to the wells, and the incubation was carried out at 37°C for 1 hour. The plates were then washed and incubated with HRP-conjugated goat anti-human IgG (H+L) (ABclonal, AS003) at 37°C for 1 hour. The substrate TMB (Beyotime, P0209) was added and allowed to react in the dark, with color development being monitored. To stop the reaction, 1N hydrochloric acid was added, and the absorbance was measured at 450 nm. For competition ELISAs, the plates were initially coated with 0.2 ug/ml recombinant mAbs at 4°C overnight and subsequently blocked with 5% defatted milk in DPBS containing 0.05% Tween-20 (DPBS-T) for 2 hours at 37°C. Simultaneously, another aliquot of recombinant mAbs was incubated with 0.2 ug/ml biotinylated RBD (BA.4/5) for 1 hour. The incubated mixtures were then added to the pre-coated ELISA plates and incubated at room temperature. Following 1 hour of incubation and three rounds of washing, the plates underwent incubation with HRP-conjugated Streptavidin (1:5000, Beyotime) for 30 minutes at 37°C. Similar to the previous procedure, TMB (Beyotime, P0209) substrate was added, and after color development in the dark, 1N hydrochloric acid was used to stop the reaction, with the absorbance being measured at 450 nm.

### ELISA-based receptor-binding inhibition assay

ELISA plates were coated overnight at 4°C with 200 ng of hACE2 protein in 100 uL of PBS per well. Subsequently, the plates were blocked with 2% defatted milk in DPBS containing 0.05% Tween-20 (DPBS-T) for 1 hour. Concurrently, recombinant monoclonal antibodies (mAbs) or patient sera, serially diluted, were incubated with the optimal dose of biotinylated SARS-CoV-2 RBD protein for 1 hour. Following this, the mixture was added to the ELISA plates and incubated at 37°C. After 1 hour of incubation and three washes, the plates underwent incubation with HRP-conjugated Streptavidin (1:5,000, Beyotime A0303) for 1 hour at 37°C. The substrate TMB (Beyotime, P0209) was then added, and the reaction was monitored in the dark for color development. To stop the reaction, 1N hydrochloric acid was added, and the absorbance was measured at 450 nm. The determination of the half-maximal inhibition concentration (IC_50_) was performed using 4-parameter logistic regression.

### Binding affinity measurement

Affinity analysis between recombinant monoclonal antibodies (mAbs) and SARS-CoV-2 Spike RBDs from the BA.4/5, XBB.1.5, XBB.1.16, and EG.5.1 lineages was conducted using the 96-Channel Ultra High Throughput Octet® RH96 system (Sartorius Corporation). In brief, mAbs (2 μg/ml) were captured by Protein A biosensors (Octet® AHC2 biosensors, Sartorius, 18-5142) in PBST containing 0.1% BSA. Following this, the captured mAbs underwent a reaction with a gradient dilution of the Spike RBDs. The solid-phase conjugates formed were subsequently subjected to dissociation analysis in a buffer containing PBST with 0.1% BSA. The obtained results were analyzed using Data Analysis 12.0 software to determine the binding rate, dissociation rate, and affinity constant.

### Pseudoviruses neutralization assay

HEK-293T cells expressing human ACE2 (293T/ACE2) were plated in 96-well plates (Corning, 3599) at a density of 20,000 cells per well. On the following day, monoclonal antibodies (mAbs) were serially diluted in complete media, combined with wild-type (WT) pseudoviruses (Vazyme) or Omicron pseudoviruses (Vazyme), and incubated for 1 h at 37°C. Subsequently, the culture media of 293T/ACE2 cells were replaced with the pre-incubated mixture of mAbs and pseudoviruses, and the cells were cultured for an additional 16 h. Luciferase activity in 293T/ACE2 cells was measured using a luciferase reporter assay kit (Vazyme, DD1201). The IC_50_ (50% inhibitory concentration) values were determined by fitting a non-linear four-parameter dose-response curve using GraphPad Prism 8.0.

### Statistical analysis

Descriptive statistics, presented as mean ± SD, were computed for continuous variables. Statistical differences between the experimental and control groups were assessed through one-way ANOVA analysis when comparing more than two groups of data. A significance threshold of P < 0.05 was applied. IC_50_ values were determined by fitting a non-linear four-parameter dose-response curve. All statistical analyses were conducted using GraphPad Prism 8.0.

## Results

### Isolation of RBD-specific memory B cells

**Figure 1:**
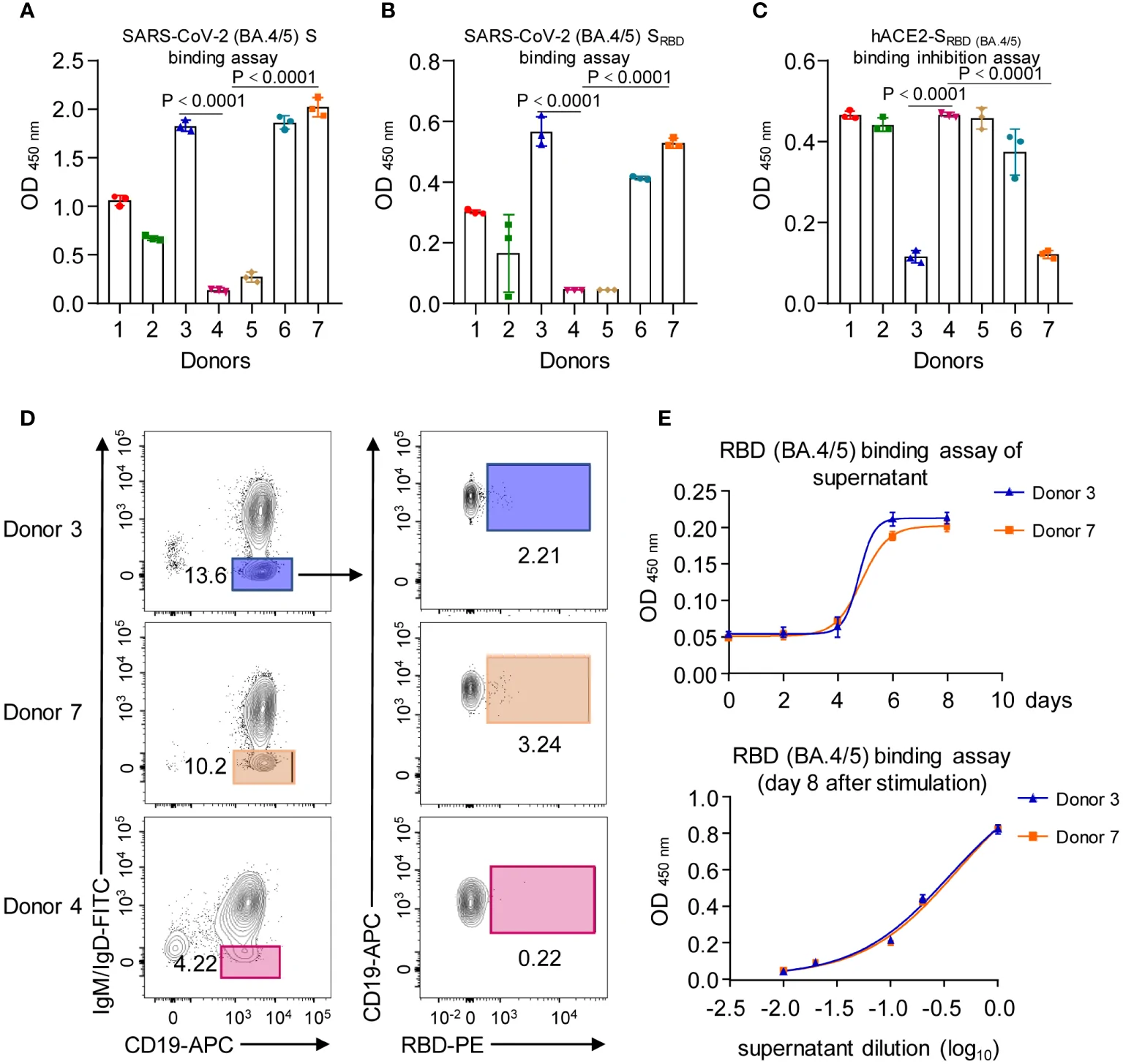
Characterization of SARS-CoV-2 donor samples. **(A)** Serum antibody reactivity for the five SARS-CoV-2 exposed patients (donor 1, 2, 3, 6 and 7) and two non-immune healthy controls (donor 4 and 5), assessed by ELISA using SARS-CoV-2 S protein (BA.4/5). **(B)** Serum antibody reactivity for the five SARS-CoV-2 exposed patients (donor 1, 2, 3, 6 and 7) and two non-immune healthy controls (donor 4 and 5), assessed by ELISA using SARS-CoV-2 S_RBD_ (BA.4/5). **(C)** Serum-mediated inhibition of SARS-CoV-2 RBD (BA.4/5) to bind with hACE2, assessed by ELISA using SARS-CoV-2 S_RBD_ (BA.4/5) and hACE2. **(D)** Gating for memory B cells in total B cells enriched by negative selection for SARS-CoV-2 exposed patients 3, 7 and healthy control 4; cells were stained with indicated antibodies and sorted by flow cytometry. **(E)** Neutralization of the SARS-CoV-2 S_RBD_ (BA.4/5) by supernatant collected from cell cultures of RBD (BA.4/5)-specific memory B cells that had been stimulated *in vitro* in human B cell expansion medium. Graphs show mean ± SD, n = 3 technical replicates for **(A, B, C, E)**.

To isolate potent mAbs against SARS-CoV-2 VOCs, we obtained blood samples from five convalescent individuals with SARS-CoV-2 Omicron infection and two healthy donors, serving as negative controls. Initially, we assessed the antigen-binding potencies of the serum from these donors. Serum antibody ELISA binding assays revealed that the serum from two healthy donors (donor 4 and 5) showed no reactivity with Omicron (BA.4/5) S protein and its Receptor Binding Domain (RBD) (**Figures 1A, B**). In contrast, serum from all five convalescent patients exhibited binding activities to both S and RBD proteins from Omicron (BA.4/5), albeit with varying affinities (**Figures 1A, B**). Next, we conducted an *in vitro* neutralization assay to evaluate the serum’s ability to inhibit RBD-hACE2 interaction. Recombinant Omicron (BA.4/5) RBD proteins were coated in ELISA plates and incubated with serum from different donors, and then combined with biotinylated recombinant hACE2 proteins. ELISA analysis indicated that among the five convalescent patients, serum from donor 3 and donor 7 significantly decreased RBD-hACE2 interaction (**Figure 1C**), suggesting the presence of neutralizing antibodies against Omicron (BA.4/5) in their blood. Further, we enriched B cells from peripheral blood mononuclear cells (PBMCs) of donor 3 and donor 7, as well as one healthy donor, using negative selection with antibody-coated magnetic beads. B cells were stained with antibodies specific for CD19, IgD, and IgM, and analytical flow cytometry was employed to assess the frequency of antigen-specific memory B cells for each donor. Class-switched memory B cells were identified by gating for an IgD^-^IgM^-^CD19^+^ population (**Figure 1D**, **Supplementary Figure S1**). From this population, we identified antigen-reactive cells using fluorophore-conjugated RBD recombinant proteins. Notably, the healthy donor did not show significant enrichment of RBD-specific memory B cells. In contrast, donor 3 and donor 7 exhibited 2.21% and 3.24% of class-switched B cells reacting with RBD antigen (**Figure 1D**). Subsequent sorting of these RBD-specific memory B cells from donor 3 and donor 7 for stimulation revealed that the secreted antibodies in the cell-culture supernatants from both donors exhibited reactivity against the RBD of Omicron (BA.4/5) (**Figure 1E**).

### Identification of single B cells secreting RBD-neutralizing antibodies

**Figure 2:**
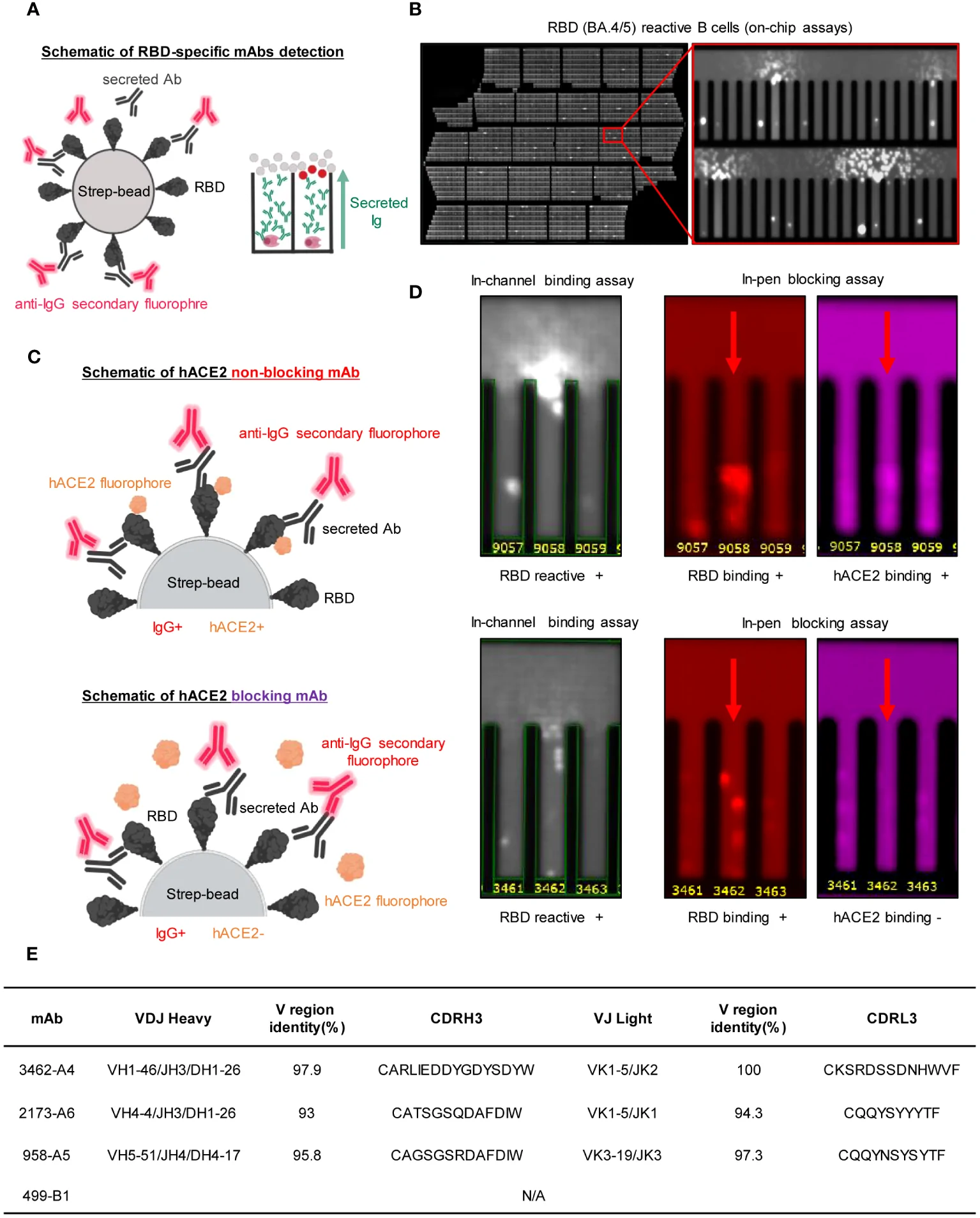
Functional assays from single antigen-reactive B cells. **(A)** Left: Biotinylated antigen (dark grey) is coupled to a streptavidin-conjugated polystyrene bead (light grey). Antibodies (Y shape in black) are secreted by single B cells loaded into individual NanoPens on the Berkeley Lights Beacon optofluidic device. Antibody binding to antigen was detected with a fluorescent anti-human IgG secondary Ab (red). Right: Schematic of fluorescing beads in the channel above a pen containing an individual B cell indicates antigen-specific reactivity. **(B)** An example of an entire 11k chip is shown, with a zoomed-in view of a section to highlight the characteristic “blooms” over pens with cells secreting RBD-reactive antibodies. **(C)** Schematic illustrating detection of secreted Ab and hACE2 binding on anRBD coated streptavidin bead. Ab binding was detected in one fluorescent channel, while hACE2 binding was detected in another fluorescent channel. The top panel illustrates an RBD-binding, non-blocking antibody, where the bead is positive for both Ab and hACE2 signals, while the bottom panel illustrates an RBD-binding mAb that competes with hACE2 for binding, where the bead is positive for only Ab signal. **(D)** Identification of mAbs with hACE2-blocking activity using single-cell functional screening. Representative images of a B cell secreting an hACE2-non-blocking mAb (top row) and a B cell secreting an hACE2-blocking mAb (bottom row). Streptavidin beads are loaded into the same pens as B cells. The fluorescence of the streptavidin beads in the same pen as the B cell secreting hACE2-blocking Abs is reduced relative to adjacent wells, indicating hACE2-blocking activity. **(E)** V(D)J gene usage, V-region identity (%), and CDR3 amino acid sequences of the heavy and light chains of the recombinant mAbs, analyzed using the IMGT database. The assay principles illustrated in panels **(A)** and **(C)** follow a previously described Beacon-based antigen-binding and hACE2-blocking workflow (64). The representative chip images shown in panels **(B)** and **(D)** were generated from the experiments reported in this study.

We loaded 8-day-activated B cells onto a Berkeley Lights Beacon optofluidic instrument in a medium promoting plasma-cell survival. The RBD reactivity of secreted antibodies from individual B cells was measured for thousands of cells (**Supplementary Figure S2A**). This high-throughput single-cell analysis demonstrated the successful loading of 4,246 cells into separate NanoPens on the chip of the Berkeley Lights Beacon optofluidic device (**Supplementary Figure S2B**). This enabled us to conduct a single-cell functional screen to identify B cells secreting RBD-reactive IgG (**Supplementary Figure S2A**). In brief, biotinylated RBD (dark gray) was conjugated to streptavidin-coupled polystyrene beads (light grey) and then loaded into the channel above the NanoPens. Single B cells secreted antibodies (Y shape in black) into the individual NanoPens, and antibody binding to RBD antigen was detected with a fluorescent anti-human IgG secondary antibody (red). As a result, reactive antibodies diffusing out of a pen were visualized as a bloom of fluorescence (**Figures 2A, B**). Through this approach, we identified 137 out of the 4,246 cells in the NanoPens producing RBD-reactive antibodies. Building upon these RBD-positive cells, we employed another strategy to screen single B cells for the ability to produce antibodies that block the human ACE2 (hACE2) receptor binding to the RBD antigen. Initially, RBD-conjugated streptavidin beads were loaded into the NanoPens to directly bind to RBD-reactive antibodies for 30 minutes. Subsequently, soluble hACE2 was loaded into the same NanoPens as the streptavidin beads to allow its binding to RBD. Antibody binding to RBD-conjugated beads was detected in one fluorescent channel, while hACE2 binding was detected in another fluorescent channel. We found that the single B cell in pen 3462 secreted RBD-binding antibodies that competed with hACE2 for binding, with the beads showing positive signals only for RBD binding (**Figures 2C, D**). In contrast, the single B cell in pen 9058 secreted RBD-binding antibodies without hACE2-blocking properties, with the beads showing positive signals for both RBD and hACE2 binding (**Figures 2C, D**). This effort led to the identification of a total of 4 single B cells that produced antibodies blocking RBD-hACE2 interaction (**Supplementary Figure S2B**).

We retrieved these four cells from the instrument and sequenced the heavy- and light-chain genes from each single B cell, which were then cloned into immunoglobulin expression vectors. While facing challenges with the cDNA amplification of the VH gene in one of the exported cells, we successfully obtained gene information encoding both VH and VL from the other three candidates, namely 958-A5, 2173-A6, and 3462-A4 (**Supplementary Figure S2C**). Using Igblast, we conducted an analysis of the CDR3 region, nucleotide sequence length, variable region identity, and germline characteristics of IgH and IgL for all three antibodies (**Figure 2E**). The results revealed that each of the three antibodies utilized a distinct heavy-chain germline. Specifically, 958-A5 belonged to the IGHV5-51 family, 2173-A6 belonged to the IGHV4-4 family, and 3462-A4 belonged to the IGHV1-46 family (**Figure 2E**). In contrast, 2173-A6 and 3462-A4 shared a light-chain germline belonging to the IGKV1-5 family, while 958-A5 employed a different light-chain germline belonging to IGLV3-19 (**Figure 2E**). Moreover, all three antibodies exhibited more than 90% identity in the variable regions when compared to their respective germline genes, both for VH and VL (**Figure 2E**). The alignment of the antibodies for the CDRH3 and CDRL3 regions is also depicted in **Figure 2E**.

### Binding activities of RBD-specific antibodies

**Figure 3:**
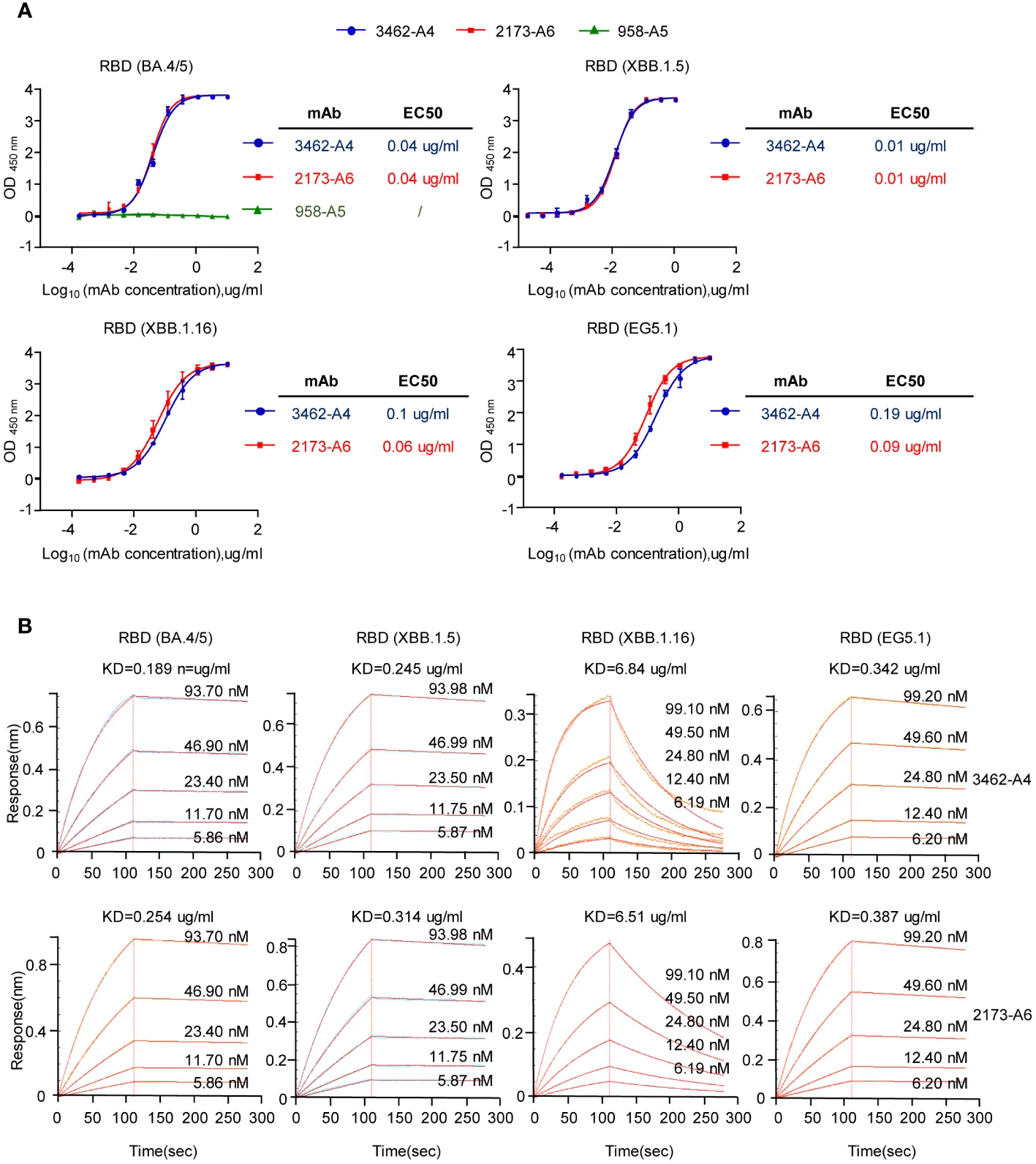
Reactivity and affinity characteristics of the mAbs to SARS-CoV-2 RBD. **(A)** ELISA analysis assessing the reactivity of the mAbs against the RBDs (BA.4/5, XBB.1.5, XBB.1.16 and EG5.1). **(B)** Binding and dissociation kinetics of the mAbs (with different concerntrations) against the RBDs (BA.4/5, XBB.1.5, XBB.1.16 and EG5.1), assessed by surface plasmon resonance (SPR). Graphs show mean ± SD, n = 3 technical replicates for **(A)**.

We expressed and purified monoclonal antibodies (mAbs) 958-A5, 2173-A6, and 3462-A4 in HEK293F cells (**Supplementary Figures S3A, B**). Since the bait for obtaining neutralizing mAbs was the RBD of SARS-CoV-2 Omicron (BA.4/5), our initial assessment focused on the binding of 2173-A6 and 3462-A4 to the RBD (BA.4/5). ELISA binding assays demonstrated effective reactions of 2173-A6 and 3462-A4 with RBD (BA.4/5) antigen, with both EC_50_ values of 0.04 ug/ml (**Figure 3A**). However, 958-A5 exhibited no binding affinities to the RBD (BA.4/5) under the same experimental conditions (**Figure 3A**). This suggests that the lack of interaction between 958-A5 and RBD (BA.4/5) may be attributed to a false-positive reaction during the functional single B cell analysis performed on the Beacon instrument. Next, we assessed 2173-A6 and 3462-A4 for their reactivity with other SARS-CoV-2 lineages. ELISA results revealed that both antibodies effectively reacted with RBDs from multiple SARS-CoV-2 Omicron lineages, including XBB.1.16, XBB.1.5, and EG.5.1, with EC_50_ values of 0.06 ug/ml, 0.01 ug/ml, and 0.09 ug/ml for 2173-A6 and 0.1 ug/ml, 0.01 ug/ml, and 0.19 ug/ml for 3462-A4 (**Figure 3A**). The real-time association and dissociation of 2173-A6 and 3462-A4 binding to the RBDs of SARS-CoV-2 Omicron lineages were monitored using a surface plasmon resonance (SPR)-based optical assay. Both antibodies exhibited similar RBD-binding affinities to each Omicron lineage (**Figure 3B**). Notably, 2173-A6 and 3462-A4 displayed fast-on/slow-off kinetics against RBDs from Omicron BA.4/5, XBB.1.5, and EG.5.1, with equilibrium dissociation constant (KD) values of 0.254 ng/ml, 0.314 ug/ml, and 0.387 ug/ml for 2173-A6 and 0.189 ug/ml, 0.245 ug/ml, and 0.342 ug/ml for 3462-A4. However, both antibodies exhibited fast-on/fast-off kinetics against RBD from Omicron XBB.1.16, with KD values of 6.51 ug/ml for 2173-A6 and 6.84 ug/ml for 3462-A4 (**Figure 3B**). Overall, these results suggest that 2173-A6 and 3462-A4 strongly react with RBDs of multiple SARS-CoV-2 Omicron lineages.

### Neutralization properties of 2173-A6 and 3462-A4 mAbs

**Figure 4:**
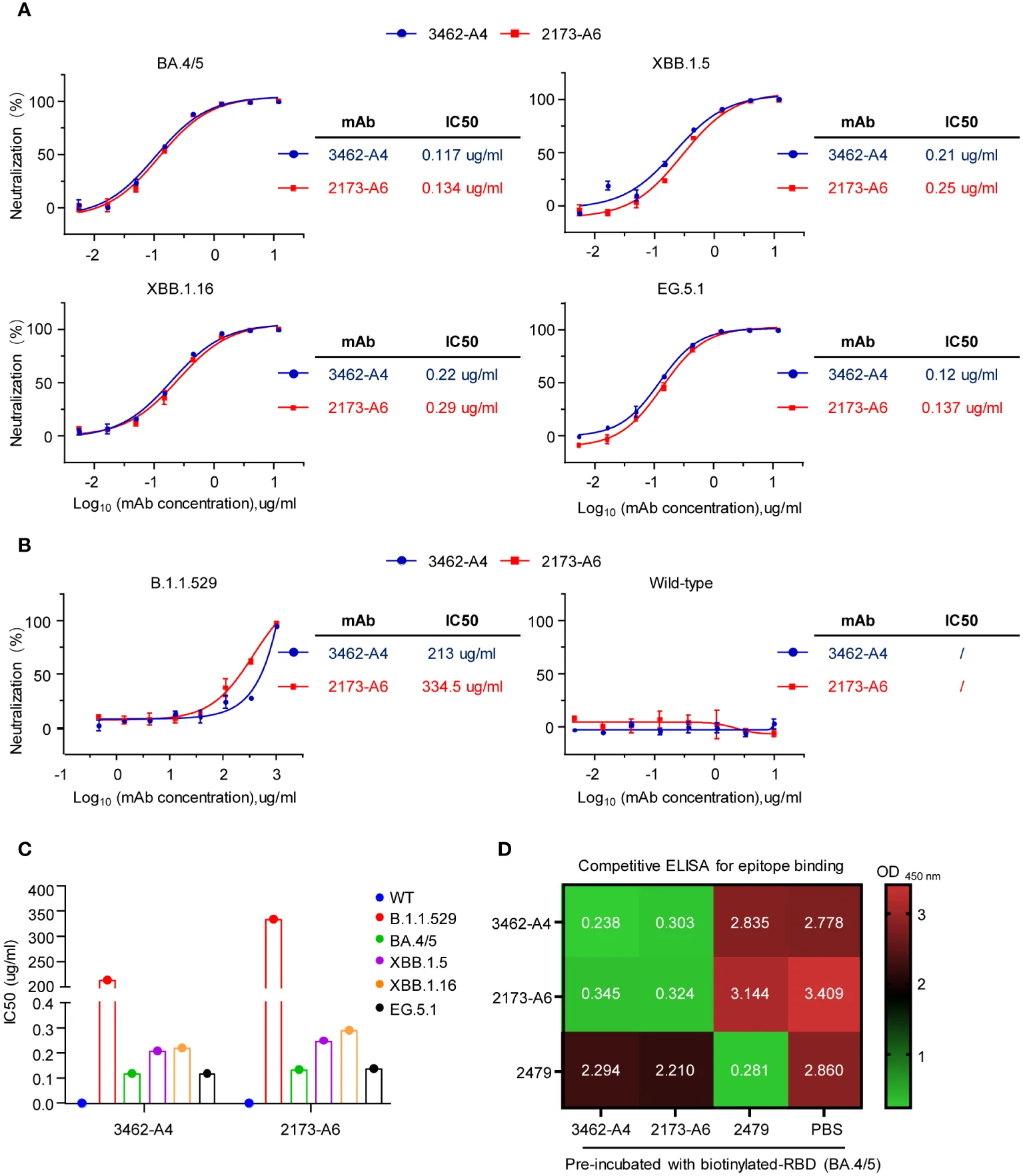
Neutralizing capacitiy of mAbs against pseudoviruses of SARS-CoV-2 **(A)** Neutralization of the mAbs to SARS-CoV-2 pseudoviruses bearing the spike of the indicated VOCs (BA.4/5, XBB.1.5, XBB.1.16 and EG.5.1). **(B)** Neutralization of the mAbs to SARS-CoV-2 pseudoviruses bearing the spike of the indicated VOCs (B1.1.529, wild type SARS-CoV-2). **(C)** Half-maximal inhibitory concentration (IC50) values of the mAbs to pseudoviruses (wild type, B1.1.529, BA.4/5, XBB.1.5, XBB.1.16 and EG.5.1). **(D)** The heat map illustrates the OD_450_ nm values obtained from the competitive ELISA analysis of the monoclonal antibodies (mAbs) for epitope binding. Graphs show mean ± SD, n = 3 technical replicates for **(A, B)**.

To assess the neutralizing capacity of 2173-A6 and 3462-A4 mAbs against SARS-CoV-2 variants, we conducted pseudovirus-based inhibition assays. The results demonstrated that both 2173-A6 and 3462-A4 efficiently neutralized a broad spectrum of Omicron variants, including BA.4/5, XBB.1.16, XBB.1.5, and EG.5.1 viruses, with IC_50_ values ranging from 0.134 ug/ml to 0.29 ug/ml for 2173-A6 and 0.117 ug/ml to 0.22 ug/ml for 3462-A4 (**Figure 4A**). Additionally, these antibodies exhibited the ability to neutralize the Omicron B1.1.529 lineage, although with reduced potency compared to other tested Omicron variants, presenting IC_50_ values of 334.5 ug/ml for 2173-A6 and 213 ug/ml for 3462-A4 (**Figures 4B, C**). However, both 2173-A6 and 3462-A4 failed to neutralize wild-type SARS-CoV-2 infection (**Figures 4B, C**). This observation suggests that the structures of the S proteins from Omicron lineages may undergo significant alterations due to mutations within their RBDs. Consequently, mAbs that effectively neutralized Omicron variants may no longer recognize the wild-type SARS-CoV-2. Considering the similar performances of 2173-A6 and 3462-A4 in various antigen reactivities (**Figures 3A, B**, **4A–C**), our next inquiry aimed to determine whether these antibodies share identical or overlapping epitopes. To explore this, we conducted a competitive ELISA and observed that 2173-A6, 3462-A4, and 2479 (a previously tested RBD (BA.4/5)-reactive but non-neutralizing antibody) efficiently bound to biotinylated-RBD (BA.4/5) antigen (**Figure 4D**). However, when the RBD (BA.4/5) was pre-treated with 2173-A6, followed by adding the mixtures to 3462-A4 precoated ELISA plates, 3462-A4 no longer reacted with RBD (BA.4/5) (**Figure 4D**). Similarly, comparable results were observed for 2173-A6 when the RBD (BA.4/5) was pre-treated with 3462-A4, followed by adding the mixtures to 2173-A6 precoated ELISA plates (**Figure 4D**). These findings suggest that 3462-A4 or 2173-A6 may target identical or at least overlapping epitopes to exert their functions. In contrast, pre-treatment with 2479 had no effect on 3462-A4 or 2173-A6 in reacting with the RBD (BA.4/5), and vice versa; pre-treatment with 3462-A4 or 2173-A6 did not affect the binding capacity of 2479 against the RBD (BA.4/5) either. This suggests that 2479 targets epitopes different from those of 3462-A4 and 2173-A6 (**Figure 4D**). In conclusion, 2173-A6 and 3462-A4 potently neutralized multiple Omicron variants of SARS-CoV-2 by binding to identical or overlapping epitopes.

## Discussion

The rapid emergence and multiple rounds of SARS-CoV-2 variants necessitate the identification of antibodies that can offer durable protection. This can be achieved by targeting conserved sites or utilizing avidity to maintain strong binding to mutated epitopes, ensuring sustained effectiveness in the face of continuing virus evolution. Despite the approval of multiple monoclonal antibodies developed during the early pandemic, frequent viral mutations triggering antigenic shifts and structural changes have led to the immune escape of SARS-CoV-2 variants, especially Omicron lineages, from most mAbs (34–38). In this study, we employed the Beacon platform and rapidly identified two mAbs (2173-A6 and 3462-A4) targeting the RBD of the S protein from individuals previously infected with the Omicron variant (**Supplementary Figure S4**). These antibodies potently and broadly neutralized pseudoviruses of multiple SARS-CoV-2 Omicron lineages, including BA.4/5, XBB.1.16, XBB.1.5, and EG.5.1. However, neither 2173-A6 nor 3462-A4 exhibited reactivity against wild-type SARS-CoV-2. It has been reported that Omicron variants harbor more than 30 mutations throughout the spike protein, with at least 15 substitutions in the RBD (14, 24, 25). The mutation-induced structural changes of RBD may explain the failure of 2173-A6 and 3462-A4 to neutralize wild-type SARS-CoV-2.

Since 2173-A6 and 3462-A4 demonstrated similar performances against SARS-CoV-2 variants, we investigated the relationship of their binding epitopes. Our competitive ELISA analysis showed that pre-treatment of the RBD (BA.4/5) with 2173-A6 substantially affected the binding of 3462-A4 to the RBD (BA.4/5) and vice versa, indicating that 2173-A6 and 3462-A4 may exert their functions by binding identical or overlapping epitopes, albeit with different VH and VL sequences. It should be noted that this study did not determine whether 2173-A6 and 3462-A4 exhibited reactivity against other SARS-CoV-2 variants, such as Alpha (B.1.1.7), Beta (B.1.351), Gamma (P.1), and Delta (B.1.617.2). Exploring the broad neutralizing potency of 2173-A6 and 3462-A4 against additional SARS-CoV-2 variants, other than Omicron lineages, would be of interest. Furthermore, the *in vivo* functions of 2173-A6 and 3462-A4 against Omicron viruses still require further verification in the future.

Human-derived neutralizing mAbs stand out as promising therapeutic agents against emerging viruses, and samples from convalescent individuals with prior viral infections offer an abundant source of mAbs for therapeutic development (43). While FACS-based single B cell isolation technology is well-established, cells obtained through this method cannot be immediately distinguished to determine which ones have the potential to secrete neutralizing antibodies until further labor-intensive assays are conducted. The Berkeley Lights Beacon system represents a breakthrough platform that employs microfluidic technologies for direct B cell antibody discovery. Integrating opto-electropositioning (OEP), microfluidics, and microscopy, this system enables single-cell manipulation, culturing, and phenotypic analysis on nanofluidic chips (59–62). Through the Beacon platform, we efficiently screened single B cells with neutralizing properties against RBD (BA.4/5) directly on chips in a high-throughput manner within hours (59–62). Cells of interest were then individually exported from the microfluidic chip for retrieving sequences encoding native VH and VL pairings of the original antibodies. Recent studies propose that non-competing antibody cocktail therapy represents a promising approach against SARS-CoV-2 variants with spike protein mutations (64). Given the ongoing mutations of SARS-CoV-2, it is particularly significant to develop neutralizing antibodies against the latest Variants of Concern (VOCs) for durable COVID-19 therapeutics in the shortest possible timeframe. The Beacon instrument offers a platform that substantially reduces the labor-intensive workflow of the antibody discovery process primarily through its capacity for high-throughput screening and single-cell analysis. It will accelerate the development of novel therapeutic antibodies for treating newly emerging SARS-CoV-2 variants and other unknown infectious diseases in the future.

While the Berkeley Beacon platform demonstrates a significant advance in rapid antibody isolation, it is crucial to discuss its limitations in the context of our findings. A crucial step in our methodology involves the culturing and activation of antigen-specific memory B cells to transform them into plasma cells, which are the source of antibody production. However, it is important to note that not all memory B cells successfully undergo this transformation. The efficiency of conversion from memory B cells to plasma cells is a key determinant in the overall yield of antibodies. This aspect, often variable and unpredictable, significantly influences the number of plasma cells capable of producing the desired neutralizing antibodies. Another important technical aspect is the limitation imposed by the nanochip used in our study. The nanochip in the Berkeley Beacon platform has a capacity of housing only 11,000 single B cells. Considering that this number represents only a fraction of our cultured total cells, the capacity of the nanochip directly limits the number of B cells that can be screened. Furthermore, it should be noted that some single B cells may not survive the on-chip assay process. Additionally, unsuccessful cDNA amplification of the VH gene from exported cells can occur, which also impacts the overall yield of successfully isolated monoclonal antibodies.

In conclusion, our study presents a novel approach to antibody isolation using the Berkeley Beacon platform. Despite the inherent biological and technical limitations, the identification of two potent monoclonal antibodies (2173-A6 and 3462-A4) highlights the platform’s potential in rapid antibody discovery. Further research and development are needed to optimize these processes for higher efficiency and broader application.

## Data availability statement

The original contributions presented in the study are included in the article/**Supplementary Materials**, further inquiries can be directed to the corresponding authors.

## Ethics statement

The studies involving humans were approved by The Ethics Committee of Chongqing International Institute for Immunology. The studies were conducted in accordance with the local legislation and institutional requirements. The participants provided their written informed consent to participate in this study.

## Author contributions

PY: Data curation, Formal Analysis, Investigation, Methodology, Software, Validation, Visualization, Writing – review & editing. JR: Data curation, Formal Analysis, Investigation, Writing – review & editing. RY: Data curation, Investigation, Methodology, Visualization, Writing – review & editing. HZ: Investigation, Validation, Writing – review & editing. SL: Investigation, Writing – review & editing. YW: Project administration, Resources, Supervision, Validation, Writing – review & editing. TZ: Conceptualization, Funding acquisition, Project administration, Resources, Supervision, Validation, Writing – review & editing. TX: Conceptualization, Funding acquisition, Project administration, Supervision, Validation, Visualization, Writing – original draft, Writing – review & editing.
