# Correction: Rapid isolation of pan-neutralizing antibodies against Omicron variants from convalescent individuals infected with SARS-CoV-2

**DOI:** 10.3389/fimmu.2026.1981857

**Published:** 2026-09-30

**Authors:** Peng Yu, Jingping Ran, Ruiqi Yang, Hang Zhu, Song Lu, Yuzhang Wu, Tingting Zhao, Tianchen Xiong

**Affiliations:** 1 Antibody Research Platform, Chongqing International Institute for Immunology, Chongqing, China; 2 School of Pharmacy and Bioengineering, Chongqing University of Technology, Chongqing, China

**Keywords:** SARS-CoV-2, COVID-19, Omicron, neutralizing antibody, beacon

Reference 65 “Zost SJ, Gilchuk P, Chen RE, Case JB, Reidy JX, Trivette A, et al. Rapid isolation and profiling of a diverse panel of human monoclonal antibodies targeting the SARS-CoV-2 spike protein. Nat Med. (2020) 26:1422-1427. doi: 10.1038/s41591-020-0998-x” was not cited in the article. The citation has now been inserted in the section **Materials and methods**, *Single RBD-specific ASCs selection by Berkeley lights beacon system*, paragraph 1 and should read:

“Activated ASCs samples were imported automatically onto OptoSelect™ 11k chips in a novel plasmablast survival medium that promotes antibody secretion and preserves cell viability (63, 65).”

There was a mistake in the caption of **Figure 2** as published. A relevant citation to a previous study describing the Beacon-based antigen-binding and hACE2-blocking assay workflow was omitted. The corrected caption of **Figure 2** appears below.

**Figure 2:**
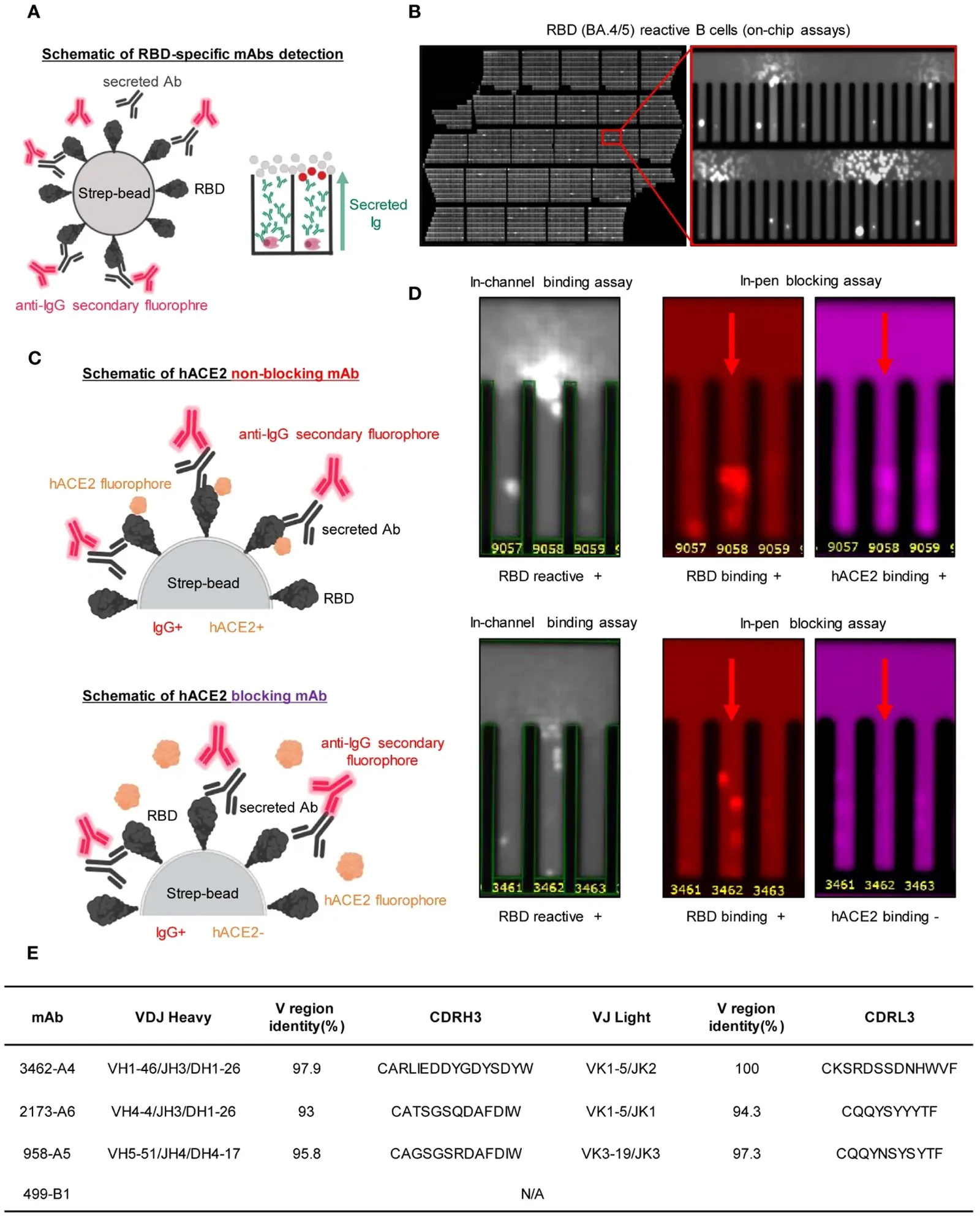
Functional assays from single antigen-reactive B cells. **(A)** Left: Biotinylated antigen (dark grey) is coupled to a streptavidin-conjugated polystyrene bead (light grey). Antibodies (Y shape in black) are secreted by single B cells loaded into individual NanoPens on the Berkeley Lights Beacon optofluidic device. Antibody binding to antigen was detected with a fluorescent anti-human IgG secondary Ab (red). Right: Schematic of fluorescing beads in the channel above a pen containing an individual B cell indicates antigen-specific reactivity. **(B)** An example of an entire 11k chip is shown, with a zoomed-in view of a section to highlight the characteristic “blooms” over pens with cells secreting RBD-reactive antibodies. **(C)** Schematic illustrating detection of secreted Ab and hACE2 binding on an RBD coated streptavidin bead. Ab binding was detected in one fluorescent channel, while hACE2 binding was detected in another fluorescent channel. The top panel illustrates an RBD-binding, non-blocking antibody, where the bead is positive for both Ab and hACE2 signals, while the bottom panel illustrates an RBD-binding mAb that competes with hACE2 for binding, where the bead is positive for only Ab signal. **(D)** Identification of mAbs with hACE2-blocking activity using single-cell functional screening.Representative images of a B cell secreting an hACE2-non-blocking mAb (top row) and a B cell secreting an hACE2-blocking mAb (bottom row). Streptavidin beads are loaded into the same pens as B cells. The fluorescence of the streptavidin beads in the same pen as the B cell secreting hACE2-blocking Abs is reduced relative to adjacent wells, indicating hACE2-blocking activity. **(E)** V(D)J gene usage, V-region identity (%), and CDR3 amino acid sequences of the heavy and light chains of the recombinant mAbs, analyzed using the IMGT database. The assay principles illustrated in **(A, C)** follow a previously described Beacon-based antigen-binding and hACE2-blocking workflow (65). The representative chip images shown in panels **(B, D)** were generated from the experiments reported in this study.

“**Figure 2** Functional assays from single antigen-reactive B cells. (A) Left: Biotinylated antigen (dark grey) is coupled to a streptavidin-conjugated polystyrene bead (light grey). Antibodies (Y shape in black) are secreted by single B cells loaded into individual NanoPens on the Berkeley Lights Beacon optofluidic device. Antibody binding to antigen was detected with a fluorescent anti-human IgG secondary Ab (red). Right: Schematic of fluorescing beads in the channel above a pen containing an individual B cell indicates antigen-specific reactivity. (B) An example of an entire 11k chip is shown, with a zoomed-in view of a section to highlight the characteristic “blooms” over pens with cells secreting RBD-reactive antibodies. (C) Schematic illustrating detection of secreted Ab and hACE2 binding on an RBD coated streptavidin bead. Ab binding was detected in one fluorescent channel, while hACE2 binding was detected in another fluorescent channel. The top panel illustrates an RBD-binding, non-blocking antibody, where the bead is positive for both Ab and hACE2 signals, while the bottom panel illustrates an RBD-binding mAb that competes with hACE2 for binding, where the bead is positive for only Ab signal. (D) Identification of mAbs with hACE2-blocking activity using single-cell functional screening. Representative images of a B cell secreting an hACE2-non-blocking mAb (top row) and a B cell secreting an hACE2-blocking mAb (bottom row). Streptavidin beads are loaded into the same pens as B cells. The fluorescence of the streptavidin beads in the same pen as the B cell secreting hACE2-blocking Abs is reduced relative to adjacent wells, indicating hACE2-blocking activity. (E) V(D)J gene usage, V-region identity (%), and CDR3 amino acid sequences of the heavy and light chains of the recombinant mAbs, analyzed using the IMGT database. The assay principles illustrated in panels A and C follow a previously described Beacon-based antigen-binding and hACE2-blocking workflow (65). The representative chip images shown in panels B and D were generated from the experiments reported in this study.”

The original version of this article has been updated.

