# Supplementary figures and images for: Rapid isolation of pan-neutralizing antibodies against Omicron variants from convalescent individuals infected with SARS-CoV-2
Source: Front Immunol. 2024 Mar 6;15:1374913. doi: 10.3389/fimmu.2024.1374913 (PMC10950932; doi:10.3389/fimmu.2024.1374913)

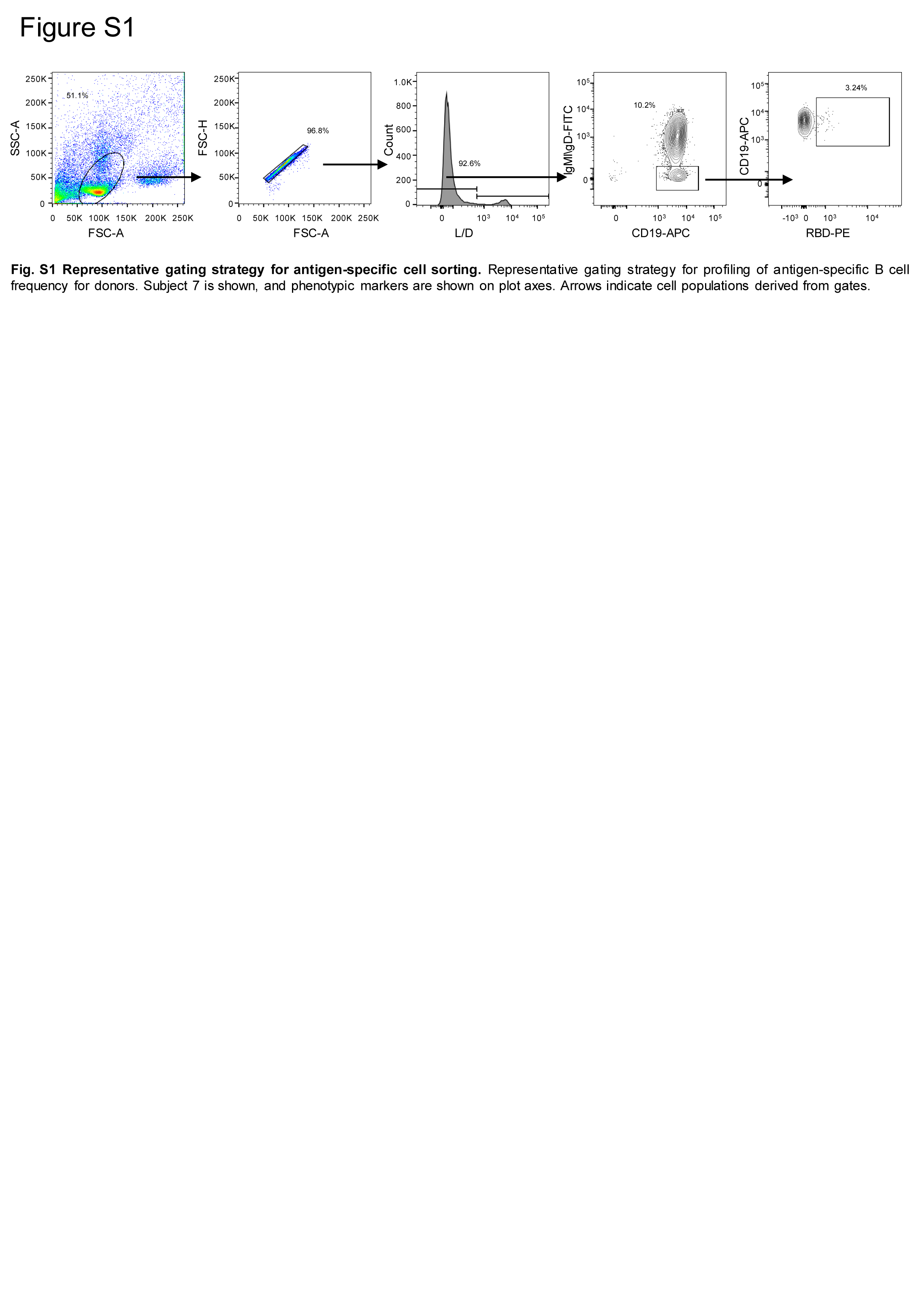

Supplement: Supplementary file 1 [file Image_1.tif]

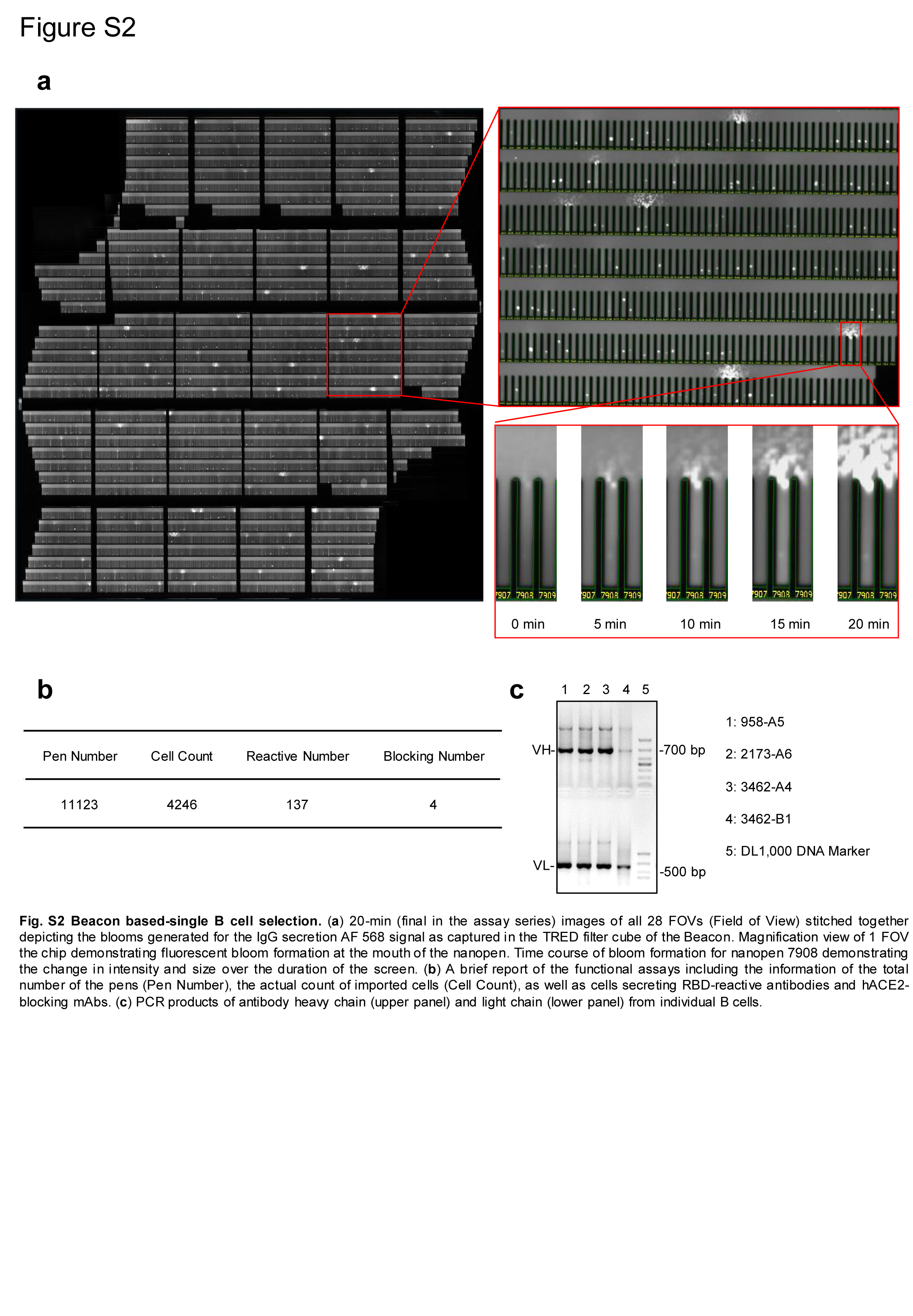

Supplement: Supplementary file 2 [file Image_2.tif]

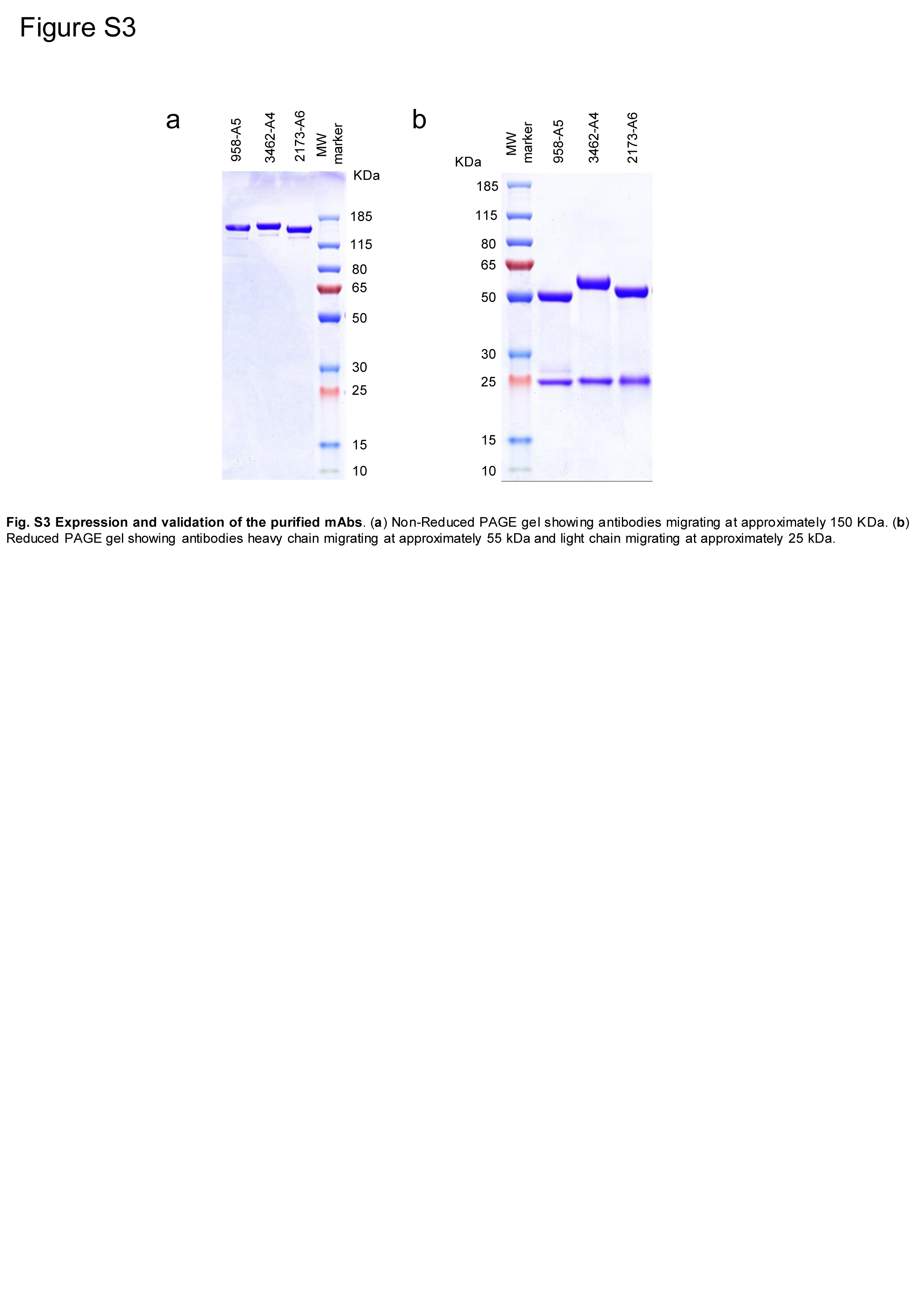

Supplement: Supplementary file 3 [file Image_3.tif]

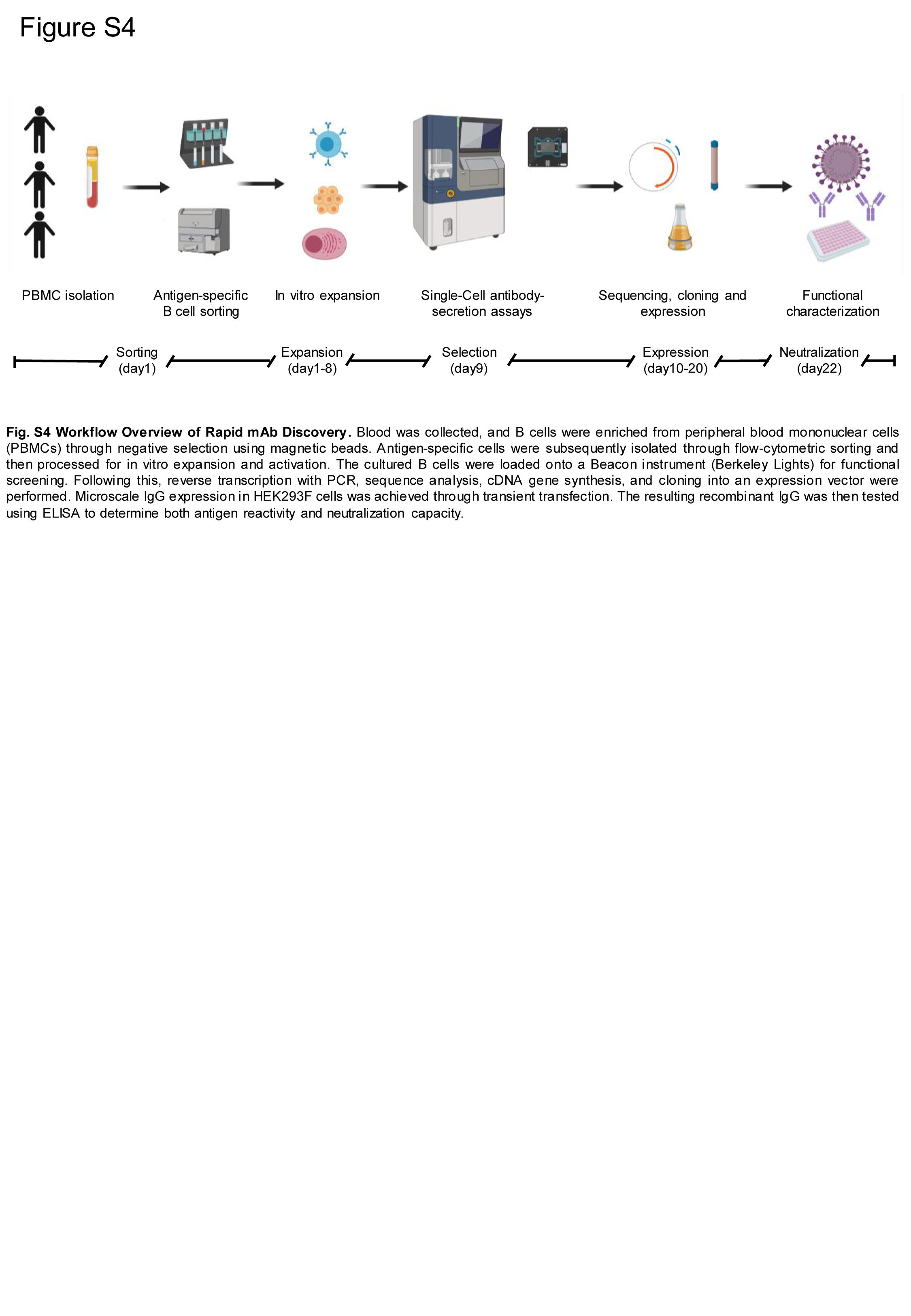

Supplement: Supplementary file 4 [file Image_4.tif]
